# The rise of registry-based research: a bibliometric analysis

**DOI:** 10.1080/17453674.2021.1937459

**Published:** 2021-06-18

**Authors:** Emilio Romanini, Irene Schettini, Marina Torre, Michele Venosa, Alessio Tarantino, Vittorio Calvisi, Gustavo Zanoli

**Affiliations:** a RomaPro Center for Hip and Knee Arthroplasty, Polo Sanitario San Feliciano, Rome, Italy;; b GLOBE, Italian Working Group on Evidence Based Orthopaedics, Rome;; c Department of Management and Law, University of Rome Tor Vergata, Rome;; d Scientific Secretariat of the Presidency, Istituto Superiore di Sanità, Rome;; e MeSVA Department, University of L’Aquila;; f Casa di Cura Santa Maria Maddalena, Occhiobello, RO, Italy

## Abstract

Background and purpose — The main purpose of arthroplasty registries is to collect information on patients, techniques, and devices to monitor and improve the outcome of the specific procedure. This study analyses the role played by registries in the orthopedic research community and describes publication trends, characteristics, and patterns of this field of research.

Patients and methods — A descriptive-bibliometric review was conducted. Scopus was the database used for the research. All articles published from 1991 to December 2020 containing keywords related to registries and arthroplasty were considered. In particular, the following dimensions were analyzed in detail: (i) papers/year; (ii) journals; (iii) countries; (iv) research growth rate; (v) collaboration among countries. VOSviewer software was used to perform the bibliometric analysis. Finally, the 50 most cited papers of the last 10 years were briefly analyzed.

Results — 3,933 articles were identified. There has been growing interest in the topic since 2010. Acta Orthopaedica ranked first for the number of articles published. The country with the largest number of articles citing registries was the United States, followed by the United Kingdom and Sweden. The relative number of articles per 100,000 inhabitants is 0.60 for Europe and 0.38 for the United States. The literature in this research area has an average yearly growth rate of 28%.

Interpretation — The publication rate in the field of arthroplasty registries is constantly growing with a noteworthy impact in the evolution of this research and clinical area. The growth rate is significantly higher than that of arthroplasty literature (28% vs. 10%) and the collaboration among countries is strong and increasing with time.

Randomized controlled trials (RCTs) are crucial in gaining knowledge regarding treatment effectiveness and supporting clinical decisions with evidence-based data. However, RCTs in orthopedic surgery present ethical, economic, and organizational challenges, therefore their number is limited and often the methodological quality is modest (Campbell et al. 2010, Mundi et al. 2014). Observational studies provide a valuable alternative method for clinical investigation in orthopedic surgery in settings in which RCTs are not feasible and when increased generalizability of findings is desired (Morshed et al. 2009, Castillo et al. 2012). Moreover, given the large amount of data collected, they offer increased power to capture rare events (e.g., complications and failure) that inadequately powered RCTs are potentially prone to miss (beta error).

Joint registries are high-quality observational, prospective cohort studies designed to collect all primary and revision cases from a specific country or geographical area, without having to rely on extrapolation from a sample. The main purpose of joint registries is to collect information on patients, implants, and procedures in order to monitor and improve the outcome of the specific procedure (Lübbeke et al. 2019). Research using data from the national registries is increasingly applied as a source of information in arthroplasty and is influencing surgical practice in many ways (Varnum et al. 2019). The arthroplasty registry community has a culture of publishing annual reports of its results (Hughes et al. 2017) but an increasing number of original studies utilizing registry data are also published in peer-reviewed journals. Moreover, many arthroplasty-related papers are referring to registry data as the basic source of information, directing research projects and resources. We evaluated the role played by registries in the orthopedic literature by means of a descriptive and bibliometric analysis of the published research and its evolution in the last 30 years. Further, we compared the growth of registry-based literature with that of the general literature on joint replacement.

## Materials and methods

We conducted a descriptive-bibliometric review in the field of arthroplasty surgery with a focus on registries. The search was initially performed on several biomedical databases and finally Scopus was selected, as it returned the largest number of published articles. All languages and all document types were considered eligible for this search. All articles published from 1979 (date of first publication of an article based on registry data) to December 2020 were considered.

Details of the search strategies are provided in Supplementary data. A quantitative-descriptive and bibliometric analysis was conducted on the final dataset obtained. In particular, we analyzed the following dimensions: (i) number of papers/year; (ii) journals; (iii) countries; (iv) research growth rate; (v) journals’ impact factor; (vi) collaboration among countries. Technical details concerning bibliometrics, i.e., methodology and software used for the analysis, can also be found in Supplementary data.

A comparison was then performed between articles in our dataset and in the larger sample obtained without restricting the search to registry-based research, to compare the growth rate of papers citing registries with those dedicated to joint replacement in general.

Moreover, complying with the common use in bibliometric literature (Lobo et al. 2020, Yakkanti et al. 2020), the 50 most cited papers related to registries were analyzed; to avoid the possibility of retrieving mostly “classical” papers, we limited the analysis to the last 10 years.

Finally, articles published on the top 5 ranked journals in general and internal medicine (“the Big Five”: NEJM, Lancet, BMJ, JAMA, Annals of Internal Medicine) were analyzed separately to offer an estimate of diffusion of registry-based research outside of the orthopedic field.

## Results

**Figure 1.**
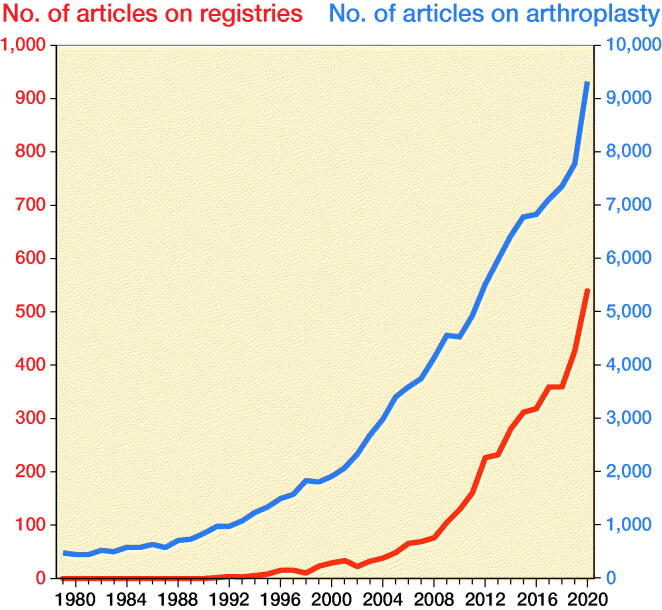
Temporal trends of articles on registries (red line) and on arthroplasty in general (blue line). Source: Authors’ elaboration from Scopus.

3,933 articles were retrieved through Scopus. The descriptive analysis showed a continuous growth of research citing the keywords related to registries throughout the entire timespan (Figure 1).

The average annual growth rate of literature concerning registries is about 30%. We compared this with the average annual growth rate of the entire literature (123,126 articles) related to joint replacement, that is about 8%.

11 journals published more than 50 papers and they are listed in the Table; Acta Orthopaedica ranked first for the number of articles published. 53 articles were published in the Big Five journals (1.5%): BMJ 25; Lancet 20; Annals of Internal Medicine 4; JAMA 4, NEJM 0.

**Table:** List of the most productive journals (> 50 articles about registries)

Source title	Number of publications 1979–2020	Impact factor 2019	5-year impact factor^a^	Country
Acta Orthopaedica	395	3.0	3.5	NOF
Journal of Arthroplasty	369	3.7	3.7	USA
Clinical Orthopaedics and Related Research	260	4.3	4.7	USA
Journal of Bone and Joint Surgery American Volume	209	4.6	5.7	USA
Bone and Joint Journal	198	4.3	4.1	England
BMC Musculoskeletal Disorders	124	1.9	2.4	England
International Orthopaedics	85	2.9	2.8	Germany
Journal of Shoulder and Elbow Surgery	78	2.8	3.3	USA
HIP International	72	1.3	1.3	Italy
Knee Surgery Sports Traumatology Arthroscopy	69	3.2	3.2	Germany
Archives of Orthopaedic and Trauma Surgery	58	2.0	2.1	Germany

a Source: Authors’ elaboration from https://jcr.clarivate.com

NOF = Nordic Orthopaedic Federation

**Figure 2.**
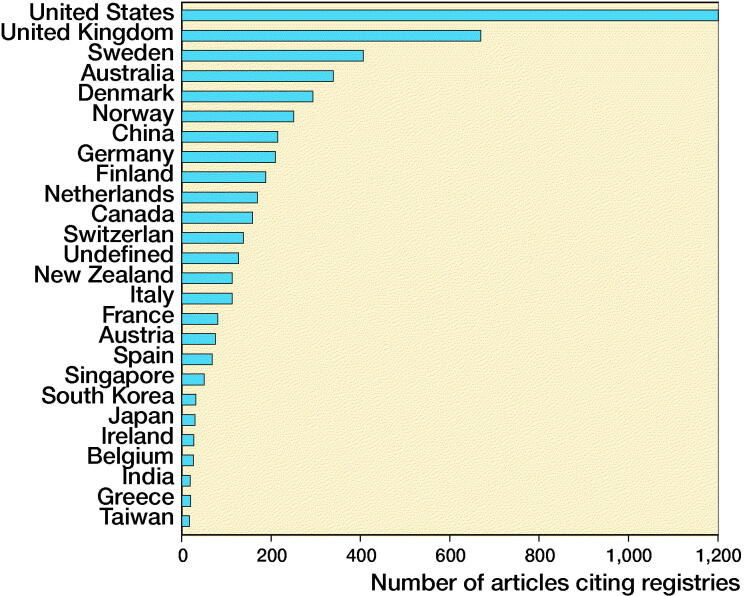
Most productive countries (Scopus). The number of articles per 100,000 inhabitants is 0.60 for Europe and 0.38 for the United States.

The country with the largest number of articles citing registries was the United States, followed by the United Kingdom and Sweden. Europe as a whole is by far the most prolific geographical area, largely due to the contribution of northern European countries that were pioneers in this type of research (Figure 2).

Among the 50 most cited papers, 39 were original studies, 11 were reviews (8 systematic, 3 narrative). In 36 papers the study was performed using original registry data, in 3 cases the analysis was performed by authors not directly involved with one specific registry. 11 studies were published in 2 of the “Big Five” journals: Lancet 7, BMJ 4.

**Figure 3.**
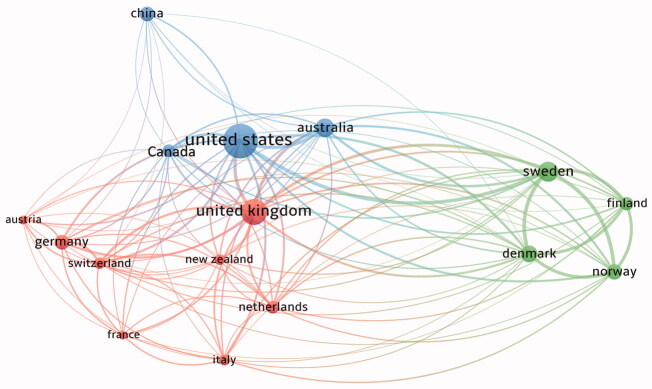
Map of the 15 most productive countries and of the relationships among the international research groups. Source: Authors’ elaboration from VOSviewer software.

The bibliometric analysis shows the collaboration among countries: the size of the label and the country circle indicates the number of citations: the higher the number of citations, the larger the circles. Distances between circles represents correlation of countries in terms of scientific collaboration links (Figure 3).

**Figure 4.**
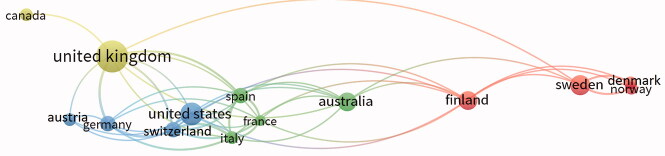
Map of the 15 most productive countries and of the relationships among the international research groups among the 50 most cited articles. Source: Authors’ elaboration from VOSviewer software.

The same analysis was conducted on the subsample of the 50 most cited papers (Figure 4).

## Discussion

Arthroplasty registries were started over 40 years ago in Sweden. Since then, many countries have adopted the concept and started national registries (Varnum et al. 2019). More recently, several organizations have been created to promote collaboration among registries and to develop international standards and harmonization of data collection, such as the International Society of Arthroplasty Registries (ISAR) (https://www.isarhome.org/), International Consortium of Orthopaedic Registries (ICOR) (Sedrakyan et al. 2011), Nordic Arthroplasty Register Association (NARA) (Van Steenbergen et al. 2021), and Network of Orthopaedic Registries of Europe (NORE) (Havelin et al. 2009, Robertsson et al. 2010, Malchau et al. 2018, Pijls et al. 2019). An integration of Registry Evidence within Cochrane Reviews has been suggested as “difficult but necessary” (Zanoli 2012), and a special interest group (COchrane Unified Group on Arthroplasty Registries) has been proposed, even though this has remained for the moment episodic and needs further methodological and “political” development.

The evaluation of scientific production by means of a bibliometric analysis is useful to provide evidence of the dissemination of registry-based knowledge within the orthopedic scientific community. In this particular area of research, no formal bibliometric analysis has been published to our knowledge, though Boyer et al. (2011) performed an interesting descriptive analysis of scientific production.

The descriptive databases analysis showed a continuous growth of research citing the keywords related to registries throughout the entire timespan, and this effect is larger than the growth observed evaluating the keywords related to joint replacement in general. It is important to underline that in the last year analyzed, 2020, 6% of articles published with keywords related to joint replacement also cite the keywords concerning registries (it was 1.5% in 2000).

Acta Orthopaedica hosted the largest number of papers, reflecting the pioneer role played by the Scandinavian countries in this research and clinical area and reaffirming a well-deserved achievement (Hailer 2015).

Despite the fact that the American Joint Replacement Registry still shows low coverage (Heckmann et al. 2019), and that only some regional/institutional registries are well established, the country with the largest number of articles on registries in our sample is the United States. This might reflect, of course, the large number of orthopedic surgeons and institutions producing research in a nation that is much more populous than any European country, where registries were started. Besides, the US-published research production is possibly explained by the large number of papers published in US-based journals, such as the Journal of Arthroplasty and Clinical Orthopaedics and Related Research. The aim of our bibliometric analysis is not to rank countries, as for instance it does not take into account the quality of published articles. A qualitative analysis of published papers was beyond the scope of this article; it is in any case well known that only few well-established registries have enough coverage and completeness to provide useful data (Herberts and Malchau 2000, Van Steenbergen et al. 2015, 2021). Our search strategy retrieves not only original reports from existing registries but also papers that discuss or quote registry data; for this reason, we do not expect to find a link between quality of registry data collection and number of published articles for each country. Again, the number of articles as calculated in our analysis reflects the interest in the subject of registries rather than the quality of original data provided. We find it reassuring that our data seems to point to an increasing attention to registry-based research even from countries where no national registry is active.

The bibliometric analysis shows an increasing number of papers collecting contributions from different countries, as a result of the international collaborative initiatives. Most of the collaboration was between Sweden and Denmark: the number of publications concerning registries that these 2 countries have co-authored is 67; Sweden has a high number of co-authored articles with the United States too (59) and the United States has a strong link with the United Kingdom (56).

## Conclusion

The increasing role played by local, regional, and national registries in the development of arthroplasty is well documented by the growing body of literature depicted by this bibliometric analysis. More recently, International Collaboration across registries at patient-level data (Ranstam et al. 2011) as well as meta-analyses (Keurentjes et al. 2014, Nieuwenhuijse et al. 2014, Paxton et al. 2018) added new research perspectives and contributed to the constant growth of scientific production and international collaboration in this field, which appears as fruitful as ever. It is hoped that the growing interest in high-quality registry research will give new strength to the “quest for phased introduction of new implants” (Nelissen et al. 2011), and ultimately will lead to improved patient care.

## Supplementary Material

Supplemental Material

## Supplementary data

The search strategy is available as supplementary data in the online version of this article, http://dx.doi.org/10.1080/17453674.2021.1937459
