# Supplementary material for: The rise of registry-based research: a bibliometric analysis
Source: Acta Orthop. 2021 Jun 18;92(5):628–32. doi: 10.1080/17453674.2021.1937459 (PMC8522812; doi:10.1080/17453674.2021.1937459)
Supplement: Supplemental Material [file IORT_A_1937459_SM3063.pdf]

Supplementary data

Search strategy:

- Database: Scopus.
- Keywords: (Arthroplasty OR Joint replacement OR Hip replacement OR Knee replacement OR shoulder replacement) AND (Registry OR Registries OR Register).
- Language: all.
- Document type: all.
- Timespan: January 1979 to December 2020.

To achieve the second aim of the study the keywords (Registry OR Registries OR Register) were omitted to allow a comparison between registry-based research and whole joint replacement literature.

Quantitative-descriptive and bibliometric analyses of the dataset obtained were conducted by using a statistical-descriptive spreadsheet tool and the VOSviewer software on the metadata downloaded from Scopus.

The aim of bibliometric analysis of the literature is to analyze the editorial and textual details of written documents to produce statistical information concerning their relationships in terms of number of articles published by country, number of citations of some selected articles, and international cooperation among researchers. Thus, this approach represents a systematic, clear, and replicable process for literature review (Diodato and Gellatly 2013, Aria and Cuccurullo 2017) that allows users to quantitatively examine the bibliographic state of the art of a topic or issue by statistically analyzing interrelated information within a dataset (composed of a sample of papers).

For the present study the software VOSviewer was used. In particular, the software generates maps based on network data and allows users to visualize and explore these maps; indeed, users can extrapolate the map figure without being able to explore it. The graphical representation of bibliometric results emphasizes the association between certain variables (e.g., countries); items are represented by their label and by default also by a circle. The size of both the label and the circle of an item is determined by the weight of the item (e.g., number of articles, number of citations). The higher the weight of an item, the larger the label and the circle of the item. The color of an item is determined by the cluster to which the item belongs. Links are represented by the lines that connect the items (VOSviewer n.d.). The greater the relationship between two variables in the sample of documents, the thicker the reference line between those variables.

Aria M, Cuccurullo C. bibliometrix: an R-tool for comprehensive science mapping analysis. J Informetr 2017; 11: 959-75.

Diodato V P, Gellatly P. Dictionary of bibliometrics. New York: Routledge, Taylor & Francis Group; 2013.

VOSviewer. VOSviewer manual [Online]. Available from: https://www.vosviewer.com/getting-started#vosviewer-manual (Accessed March 29, 2020).
